# Possibilities of Broadband Power Line Communications for Smart Home and Smart Building Applications

**DOI:** 10.3390/s21010240

**Published:** 2021-01-01

**Authors:** Petr Mlýnek, Martin Rusz, Lukáš Benešl, Ján Sláčik, Petr Musil

**Affiliations:** Department of Telecommunications, Brno University of Technology, Technicka 12, 61600 Brno, Czech Republic; xruszm00@stud.feec.vutbr.cz (M.R.); xbenes44@stud.feec.vutbr.cz (L.B.); xslaci00@stud.feec.vutbr.cz (J.S.); xmusil56@stud.feec.vutbr.cz (P.M.)

**Keywords:** broadband over power line, power line communication, laboratory measurement, field measurement, performance comparison

## Abstract

Broadband Power Line communication is considered as one of possible communication technologies for the buildings communication infrastructure in the concept of Smart Building. The possible applications where BPL (Broadband over Power Lines) solution can be used for communication in the concept of Smart Building are Power Quality (PQ) measurement, Electric Vehicle or Micro Grids and Distribution Generation (DG). This article should help to determine clear performance possibilities of BPL for an implementation in Smart Building especially due to a large amount of overhead caused by cybersecurity and the protocol overhead. The possibilities of BPL were measured with five different BPL solutions. The results show a sufficient throughput on the application layer for Smart Building application, because, in the literature, various throughput limits are introduced. According to related work, there are missing measurements on the application layer for laboratory conditions as well as compared with real field measurements. In this article, we also exploit our novel idea of a broadband PLC (Power Line Communication) modem integrated into an electrical outlet.

## 1. Introduction

In recent years, with the development and availability of technology, the concepts of Smart Building, Smart Cities and Industry 4.0 have become more and more widespread. The Smart Building environment is characterized by the control and operation of home appliances, heating or air conditioning, as well as the signaling of unexpected events and the correct response of the system to this event. Everything aims to increase energy efficiency and reduce emissions [1,2,3].

One of the possible technologies suitable for this type of application is Power Line Communication (PLC). PLC uses existing power line wiring for the transmission of the information signal on the carrier frequency of low voltage (LV), medium voltage (MV) or high voltage (HV). PLC is the technology that can be compared to wireless solutions in terms of the cost of building a communication infrastructure, because power lines are already built and are available everywhere. Thus, the main advantage is the saving of funds for building a communication infrastructure. PLC technology can be divided into two basic variants [4]:

- **Narrowband PLC**: This technology operates in the 3–500 kHz frequency band, which includes the European CENELEC band 3–148.5 kHz, the US FCC band 9–500 kHz, the Chinese band 3–500 kHz and the Japanese ARIB band 10–450 kHz [5,6]. According to the data bit rate, this technology can be further divided into:
  - Low Data Rate (LDR): These are technologies with a single carrier and a data rate of several kbps. Typical examples of LDR NB-PLC are LonWorks standards, IEC 61334, X10, HomePlug C&C and SITRED.
  - High Data Rate (HDR): These are multi-carrier technologies with data rates from tens of kbps to 500 kbps. Typical examples are technologies based on ITU-T standards by G.hn, IEEE P1901.2, PRIME and G3-PLC.
- **Broadband PLC**: Broadband technology operates in the 1.8–500 MHz frequency band and features data rates at the physical layer from a few Mbps to Gbps. It is sometimes also referred to as Broadband over Power Lines (BPL). Broadband technology standards are covered by several organizations such as Universal Powerline Association (UPA), Open PLC European Research Alliance (OPERA), Consumer Electronics Power line Communication Alliance (CEPCA), Institute of Electrical and Electronics Engineers (IEEE), International Telecommunication Union (ITUT-T) and HomePlug Powerline Alliance.
  The biggest difference between the standards of individual organizations is mainly in the methods of access to the shared medium, methods of encryption and robustness of transmission. The used frequency bands, modulations and the injection of the useful signal into the electrical network are almost identical across the standards.

To ensure support for all protocols used in Smart Building applications, as well as the possibility of sufficient communication security, the broadband PLC technology is being considered for the Smart Building concept. It provides a greater throughput at the expense of a shorter communication range. Throughput, efficiency, packet loss, interference immunity and communication range are the key parameters that need to be determined in the deployment of this technology.

There is no doubt that deployment of BPL networks is an alternative or complement to technologies such as Long Term Evolution (LTE), 5G, Digital Subscriber Line (DSL) or fiber optic. There are huge deployments based on BPL communication for energy consumption and energy bills savings, battery management of loads and flexible energy tariffs. For example, the largest deployment was in Germany in Modellstadt Mannheim with 3000 homes [7]. Another deployment of BPL with 12,000 electricity and water meters was Kahramaaa’s in Qatar [8]. E.ON (name of electric utility company) is in the process of a 200,000 home roll-out based on BPL [9]. The main applications based on BPL in Smart Building are:

**Power Quality**: The distributed power system has been increased with solar and wind power local facilities, which make the grid more heterogeneous and difficult to be controlled. Thanks to power monitoring systems measuring at many different places through PLC without any additional communication lines could allow a continuous monitoring of the PQ (Power Quality) [10]. A huge issue is also monitoring of power quality in a building complex of a critical infrastructure (e.g., a hospital complex). BPL is a suitable and complementary solution to LTE for connection of a PQ monitor measuring according to EN 332000–7–710 and EN 50160. Connection of BPL and a PQ monitor provides a system which ensures increasing of power supply quality, continuity of transition to backup power supply in case of natural disasters, fluctuations in the energy network or other abnormal influences. This solution is important especially for critical infrastructure facilities, whose longer disconnection from the network can have fatal health consequences [11].

**Electric Vehicle**: The EV (Electric Vehicle) charging uses PLC to manage the communication between the vehicle and the charging post [12] based on the Combined Charging System (CCS) specification [13]. HomePlug AV or HomePlug Green are considered in CCS deployments. Nowadays, there is for example a huge deployment of the Ionity company based on CCS with HomePlug Green [14].

**Micro Grid and Distribution Generation**: Typical future smart Commercial Buildings will be equipped with renewable sources, such as solar panels, small wind turbines, natural gas cells, energy storage, battery systems and combined heat and power to allow the users to generate electricity to the grid. In addition, emergency backup generation, capability of demand response and HVAC (Heating, ventilation and air conditioning) will be considered [15,16,17]. These Micro Grid and Distribution Generation (DG) required new advanced applications with more bandwidth and higher data rates and fulfilling security requirements.

The key questions for a discussion about Smart Building based on BPL are the following:

- Is the throughput on the application layer sufficient for the applications in discussion?
- Is the throughput on the application layer sufficient for the cyber security requirements?
- Will the throughput on the application layer be sufficient for the applications in discussion for the worst case scenario?
- Will the throughput on the application layer be sufficient for fulfilling the standard TR 61850–90–12 (Wide area network engineering guidelines)? (Note that IEC 61850 and TR 61850–90–12 provide the average throughput of up to 2048 kbps. Furthermore, this standard provides the required availability of 99.9% and the minimum delay limit was set at 1000 ms for one transmission direction.)
- Could BPL be considered as communication technology for monitoring of the power quality in the building complex of a critical infrastructure (e.g., a hospital complex)?

The article is divided as follows. In the first part, Section 4, we propose the reproducible and repeatable methodology and introduce a test bed. In the second part, Section 5 and Section 6, the results of measurements and performance tests for the laboratory environment and the real field test are introduced. Finally, a comparison of different technologies is carried out and the summary and discussion are presented.

## 2. Related Works

For real implementation and roll-outs of BPL in Smart Home and Smart Building, it is not commonly known whether the throughput of BPL is guaranteed to be sufficient for the applications in discussion. From the point of view of new applications for DG management, EVs and battery chargers and with the bandwidth necessary for keeping personal data secure, it is necessary to know the exact values of the throughput for a stable and robust (against interference) BPL communication.

Many researchers conducted their studies on BPL throughput in terms of its PHY (physical layer) throughput, but very few of them focused on the exact conditions or the worst-case scenario and application throughput.

Of the published works about BPL throughput on PHY or application layers, three types of methods were recognized: laboratory, simulations and real measurements. These types of methods are used separately. Our research focuses only on comparison of laboratory measurements with real measurements in the field.

An overview of related works is summarized in Table 1 and deeply discussed in the following paragraph. According to related works summarized in Table 1, there are several results of PHY throughput measurements, laboratory measurement and throughput simulation. The laboratory measurement together with field measurement for application throughput is missing. Therefore, this article focuses on throughput measurement on the application layer for laboratory topology together with field measurement for verification of laboratory results.

López et al. [18] summarized BPL standards from DS2 to current Gh.n and IEEE 1901 and introduced the theoretical PHY throughput for ideal conditions. In [19], measurements with Corinex AV 200 (PHY throughput 200 Mbps) was carried out with the result of throughout 12 Mbps without filters and 26 Mbps with filters for in-home scenario with typical appliances (TV, PC, refrigerator). Horvat et al. [20] described laboratory measurements with PLC adapters with the HomePlug AV standard. At a distance of 50 m, the adapters reached an average throughput of 30–35 Mbps. Castor et al. [21] used BB-PLC modems for onshore oil and gas industry applications on MV line. Modems worked in the spectrum 2–32 MHz, but the standard is not defined in the article. The throughout is 50–120 Mbps. Tomimura and Neto [22] described field measurements on a low voltage overhead power line with a length of 240 m using an adapter with the HomePlug AV standard. The throughput on the transport layer was in the range of 5.8–21 Mbps. The authors of [23] simulated the topology of a distribution network in which the Carrier-Sense Multiple Access (CSMA) is commonly used. In the simulation, they proposed an improved adaptive p-persistent CSMA protocol based on the dynamic game optimization. The average bandwidth usage is improved by 89.2%. The throughput in the simulation is up to 1 Mbps. Sangsuwan et al. [24] also focused on laboratory measurements of throughput. The measurement was performed at a distance of 100 m with and without interference. Lee et al. [25] presented simulation and real measurements. The measurements were performed at a point-to-point distance of 2–70 feet and the range was 1.6–5.2 Mbps on the HomePlug AV standard. In [26], approximately 45–100 Mbps was measured between distribution stations in the 8–34 MHz frequency range and 20–65 Mbps at the frequency of 2–12 MHz in a building. Schwager et al. [27] presented a simulation of a potential broadband PLC system with the frequency range 4–30 MHz, 1728 carriers, Forward Error Correction (FEC) coding and with Additive White Gaussian Noise (AWGN) at 26 MHz. The result of the simulation is PHY throughput 142.9 Mbps with 64QAM (Quadrature Amplitude Modulation) 190.6 Mbps (256QAM) and 238.2 Mbps (1024QAM). Piñero et al. [28] presented a simulated comparison between the standard HomePlug AV and HomePlug AV with the Greatest Common Tonemap (GCT) algorithm, which is a simple multicast algorithm occasionally used in wireless networks. Throughput in the topology with ten clients was 10 Mbps with HomePlug AV and 75 Mbps with used GCT algorithm. Statistical evaluation of 55 million PLC channels and topology measurements by more than 75,000 end users was described by Weling and Nazari [29]. The largest drop in throughput to 11 Mbps was at approximately 6 PM. This may be due to the switching of a large number of electrical devices, such as televisions and lamps. Sasikumar and Narayanan [30] described the simulation and the advantages of incremental bit reading with a multiwire PLC. Better performance in terms of the coarse bit rate was achieved with multi-wire bit-read PLCs, rather than with two-wire bit-read PLCs. Maximum transmission speeds of around 180 Mbps and 230 Mbps were obtained for two-wire and multiwire PLCs with a transmission power of −55 dBm/Hz. Real measurements from the city of Juiz de Fora in Brazil [31] showed a throughput of 1.23–5.15 Mbps. The connection can be established only in a distance of 300 m. Merkulov and Shuvalov [32] presented a practical example of a HomePlug AV modem application in the unique task of IP video signals transmission through a power line with the length of more than 700 m. The Transmission Control Protocol (TCP) throughput of the BPL adapter fluctuated within 800–900 Mbps. The maximum speed guaranteed by the manufacturer is up to 1200 Mbps [33]. Mizutani et al. [34] described measurements performed using broadband modems with the HD-PLC (High Definition Power Line Communication) standard with a maximum throughput of 220 Mbps. The result of measurements in home electrical network was a throughput over 63 Mbps at 50% of sockets in the house. Orgon et al. [35] presented the results of throughput measurements using a modem with the HP AV2 standard in real home electrical network. In a topology with connected appliances, the installed modems reached transmission speeds of 622–766 Mbps. Osman et al. [36] focused on experimental measurements in a real home installation in Malaysia. They used modems with the HomePlug AV standard and iPerf3 software. The result of this measurement is an average throughput of 95.14 Mbps between modems on the same phase with a throughput deviation of 62%. The simulations performed by Arab et al. [37] focus on quality of services testing. Testing took place in several different scenarios with different parameters set. The best results were achieved with the acknowledge timeout parameter set at 200 us and the final throughput was 22 Mbps. Hallak et al. [38] presented measurements with a BPL modem, which are part of the ITU-T G.hn standard. The transmission speed reaches 95.5 Mbps; in the case of using a repeater, the speed is reduced to 48 Mbps. The IEEE Std 1901–2010 [39] standard defines high-speed communication equipment through power lines of more than 100 Mbps at the physical layer. At the same time, transmission frequency is up to 100 MHz. Mahmood et al. [41] described the key issues associated with broadband communication, including attenuation, interference and reflection, noise and safety. Attenuation has a big impact on communication; the signal power is decreased when increasing the distance. The signal is attenuated with increased distance and frequency, while the cable loss causes an increase in signal attenuation. There are many different signals on power line networks, but, for communications, all signals except communications signals are considered interference or noise. Noise is one of the most important challenges facing PLC technology, where noise occurs from internal and external sources. In the end, there is security. It also needs to be addressed, even though the PLC technology is located on the power line. Another use of PLC technology can be in the principle of partial discharge detection. The authors of [42,43] pointed out the effect of a partial discharge on physical speed. In the normal state, the physical speed was approximately 170 Mbps; when partial discharges were injected, the speed dropped to 100 Mbps. The technology can be used as a diagnostic tool.

## 3. Motivation and Goals

According to results of achievable throughput analyzed in Section 2, the performance possibilities of BPL for implementation in Smart Building are not clear. New advanced applications, such as DG management and EVs or battery chargers, require more bandwidth and higher data rates. New security requirements also require more bandwidth to fulfill security implementations necessary for operations connected to payment and billing (e.g., EV applications), critical infrastructure (remote switching, for example smart meter disconnection) or according to the GDPR (General Data Protection Regulation) law (essential data of users). The main motivation of the article is to introduce exact values of performance and other Quality of Services (QoS) parameters for the consideration of implementing BPL technology in the Smart Building. The novelty of the research introduced in the article is also a new design of the BPL model integrated in a power plug. The integrated BPL model is designed directly for Smart Homes and Smart Building where there is a problem with space and also the need for a nice look.

The main goals of the article are the following:

- Introduce possible throughput of different BPL solutions for different conditions (noise and attenuation).
- Provide measurements and a repeatable methodology for new BPL solutions and the research community.
- Compare laboratory results with real field measurements.
- Provide throughput on the application layer, as the PLC network performance is usually described using the phrase “up to” or introduced on the physical layer.
- Provide throughput for the “worst case“ scenario with high noise level.
- Define and quantify high data rates and high-level security.
- Present a BPL modem integrated into power plug.

*Why BPL and why high bandwidth requirement?*

BPL and high bandwidth requirements are considered due to a large amount of overhead caused by cybersecurity and protocol overhead. For example, in the BPL rollout in Germany (Mannheim model city based on BPL communication [7]), it is mandatory to consider British Standards Institution (BSI) security requirements with TLS (Transport Layer Security). TLS requires 4–7 KB for connection establishment and 21 B for every packet [44].

TLS together with consideration of the DLMS/COSEM (Device Language Message Specification/Companion Specification for Energy Metering) provide for the register value of data transfer for billing (register size of 2 B) the total amount of approximately 2 KB of overhead of all OSI (Open Systems Interconnection) layers and security issues [45].

## 4. Methodology

The Request for Comments (RFC) 6349 methodology was considered for easy replication in different laboratories or different PLC devices. In addition, RFC 6349 represents TCP based measurements which are necessary for TLS connections. In comparison with different methods (e.g., a TCP throughput methodology is also available in EXFO FTB–Pro testers), RFC 6349 provides lower throughput, because this method considers the worst condition on the power line (the windows size was set-up according to RTT (Round-Trip Time) measured before transmission). RFC 6349 also considers TCP efficiency which counts re-connection or new establishment of TCP connection.

The RFC 6349 methodology is composed of the following phases:

- The Maximum Transmission Unit (MTU) of the line detection
- RTT measurements and calculation of the optimal window for the TCP protocol
- TCP throughput testing, TCP efficiency (how many bytes were re-sent) testing and delay of buffer (how many times did the RTT increased) testing

## 5. Field Measurement

Real testing was performed in cooperation with the company E.ON (Brno, Czech Republic), which is a distributor of electricity in the Czech Republic. Testing was performed using BPL modems, which are based on the IEEE 1901 standard and use the 2–30 MHz frequency band with FFT OFDM (Fast Fourier Transform Orthogonal Frequency-Division Multiplexing) on the physical layer. The tested topology was a direct connection of two transformer stations. The distance between the stations was 105 m. The connection took place via an underground cable line of the AYKY (cable made of aluminum for fixed installation) type with a cross-section of 185 mm^2^. The operating low voltage of the three-phase distribution was 0.4 kV. According to Geographic Information System (GIS), it was found that the cabling was installed in 1992. An overview of the parameters is given in Table 2.

**Figure 1:**
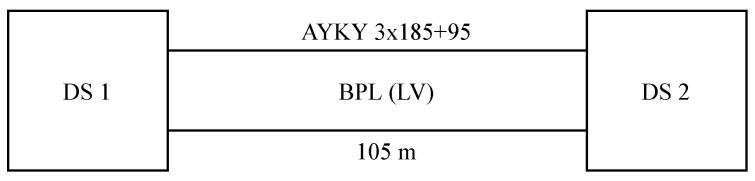
Diagram of measured topology.

The measurement procedure first defines the topology (Figure 1) that also contains the route parameters (line length, cable type, age, etc.). It is also necessary to take into account the measurement time, due to the nature of the communication technology. As part of the measurement, short-term tests were performed, where the duration of the measurement was always 10 min in the approximate time between 9 AM and 11 AM.

EXFO FTB–Pro instruments were always used for the measurements. They were connected to the BPL modem via the Ethernet interface. RFC 6349 and TCP throughput measurement methodologies were used. The achieved values of throughput (transmission rate at a given communication layer) are always the average value for the entire measurement period.

Two methods were considered for measurement:

- RFC 6349: The main advantage of the Internet Engineering Task Force (IETF) method RFC 6349 (referred to as RFC 6349) is the fact that it uses the TCP protocol for the measurement itself, which is now predominantly used for non-real-time communication on the Internet.

- TCP throughput: The main advantage of this methodology is an algorithm that works with the so-called TCP window size, which it adapts during the entire test. In the case of IETF RFC 6349, the size of the TCP window is determined when the test is initialized.

The TCP throughput testing methodology uses a reliable TCP protocol for testing, similar to RFC 6349. The main difference compared to RFC 6349 is that the size of the TCP window can be defined in the allowed range (set by the tester). The methodology is intended to allow more accurate throughput testing, due to more flexible work with the TCP window size, but, for BPL/PLC variable behavior (throughput fluctuations), this leads to extreme jumps (window size of hundreds B to MB). TCP throughput uses an algorithm that works with the size of the TCP window, which it adapts throughout the test.

RFC 6349 specifies the size of the TCP window when the test is initialized. This window determines the amount of data that can be transferred. Its size is therefore always related to the capacity of the network, which, as a rule, it must not exceed due to error-free and efficient message delivery. Furthermore, a reception window is used, which must be optimal with respect to the performance of the receiver, in order to process the received data. Proper control of this window can therefore significantly affect the results of the measured communication.

Table 3 summarizes communication directions L→R (local to remote) and R→L (remote to local); TCP window indicates the total maximum window followed by the number of connections and KB per connection in parenthesis as “(n conn.@ n KB)”; Ideal L4 means the ideal TCP throughput metric; Actual L4 shows the average of the actual TCP throughput metric the achieved with the Transmission Control Protocol/Internet Protocol (TCP/IP) protocol using the RFC 6349 methodology; TCP efficiency indicates the metric based on transmitted and re-transmitted bytes; Buffer Delay indicates the percentage metric which represents the increase in RTT during a TCP Throughput test versus the Minimum RTT; and Minimum RTT is the minimum time between the first bit of a segment sent and the last bit of the corresponding acknowledgement.

The average throughput determined by RFC 6349 was 22.2 Mbps and in the opposite direction 20.9 Mbps. Compared to the TCP throughput methodology, a higher throughput was achieved, which reached 33.375 Mbps for both directions and with the average RTT response of 46 ms. The difference in throughput is due to the different behavior of the TCP window size. With the RFC 6349 methodology, the value of the window size is determined before the measurement itself, but the TCP throughput methodology adjusts the window size as needed during the measurement. For this reason, higher values are achieved.

The results show very high values of TCP throughput. This is due to the high-quality cable connection, which was built in 1992. The cable did not degrade during that time, nor did moisture enter it. In addition, the cable does not contain any connectors or taps that could have an adverse effect on the communication.

There are not many similar measurements. Hofer [46] measured in three different TCP window sizes using the iPerf3 tool. The topology was then point-to-point. Testing in the field produced results with a median of 11.614 Mbps between 700 B and 1300 B.

## 6. Laboratory Measurement

Laboratory measurements dealt with testing of selected broadband PLC adapters according to RFC 6349 method. The principle of testing RFC 6349 testing was described in the Section 4. PLC adapters from different manufacturers with different throughput, frequency range, integrated circuits and different standards were considered for testing. Table 4 shows the list of used adapters.

### 6.1. Topology Of Measurement

**Figure 2:**
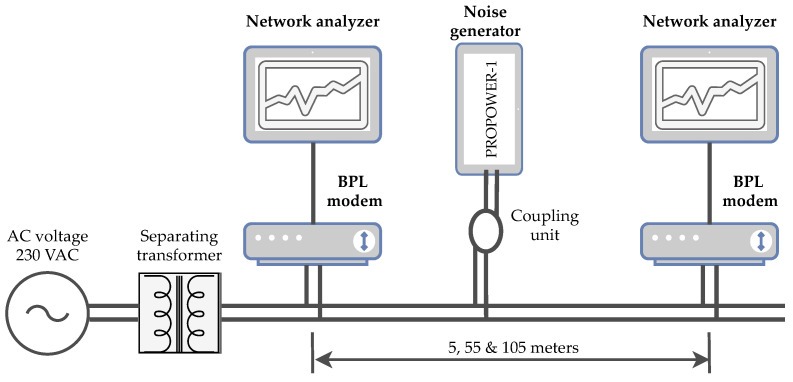
Topology for laboratory measurement.

The topology created for laboratory measurements is shown in Figure 2. This topology provides a repeatable environment for easy, time-efficient and low cost evaluation of different BPL solutions. The measured broadband modems were connected through a separated and isolated 230 V_*AC*_ electrical network. The measurements were performed at distances of 5, 55 and 105 m for all tested modems. Network analyzers EXFO FTB-1 PRO were used for testing according to the RFC 6349 methodology. The RFC 6349 was considered for simple guidelines, which ensure repeatable methodology for new BPL solutions or for comparison of our results by the research community.

Broadband noise from the PROMAX PROPOWER-1 generator was also injected into the transmission path using the coupler. The basic parameters of this generator are shown in Table 5. The frequency range of this generator is 1–50 MHz, but the noise distribution is not uniform in the whole frequency spectrum and reaches its highest noise values in the range of 2–20, 28–35 and 43–50 MHz. For laboratory measurements, the power of the generator was set to 5% corresponds to the generator switched on, 50% to half the generator power and 100% to the full generator power, which, according to the values measured by the spectrum analyzer, correspond to noise powers of 3, 5 and 11 dB. The setting of the three noise levels corresponds to conditions with low, medium and high levels of interference. From the topology point of view, the whole measurement took place in three scenarios:

- Cable route length 5 m—noise generator power level 5%, 50% and 100%.
- Cable route length 55 m–noise generator power level 5%, 50% and 100%.
- Cable route length 105 m—noise generator power level 5%, 50% and 100%.

The measurement was performed with the following rules:

- When the distance between modems changed, the connection was always re-established and another measurement was then performed.
- Noise performance increases always after measuring the previous value.
- After increasing the noise value, there was 30 s of waiting for the connection stabilization.

The main parameters that have an impact on communication in the test topology:

- Distance between tested modems
- Frequency bandwidth of tested modems
- The amount of interference noise in the transmission path

### 6.2. Results According to RFC 6349

**Figure 3:**
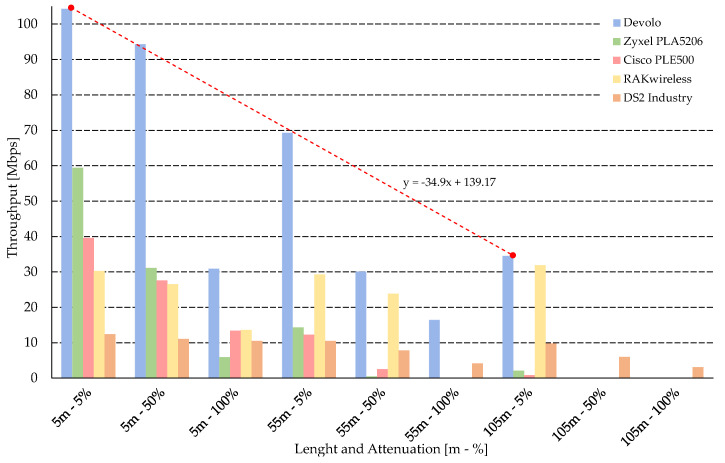
Comparison of RFC 6349–TCP throughput.

Figure 3 shows throughput on L4 layer for TCP protocol and different scenarios. This throughput could be considered as an application throughput. The application data rate is significantly lower than the physical data rate. This statement is confirmed with the results shown in Figure 3, where the measured throughout is 10 times lower than the PHY throughput presented by vendors in Table 4. An example of a decrease in throughput depending on the distance between modems is represented by a trend line for Devolo modems with a set noise output of 5%. Figure 3 also shows the impact of a particular noise scenario on throughput, when the noise level of 5 dB caused a loss of connection. This noise could be caused in real network with by switching sources with short time duration. This short time disconnection always causes a transmission re-connection and a security re-connection and provides huge delay and data loses.

As expected (standard G.hn, the largest band, declared the highest speed on the physical layer), Devolo delivered the highest speed, which is intended for home use, where speed is the key. Compared to older solutions for home use (Cisco, Zyxel, RAK), it also achieves higher resistance to interference, which can be due to the wider band, where the noise generator operates only up to 50 MHz and Devolo uses the band up to 100 MHz.

The noise immunity suggests an industry solution, which uses frequency profiles with attenuation estimation, Signal-to-Noise Ratio (SNR) estimation and calculation of transmission bits for particular carrier frequencies. Thanks to these methods, the noisy frequencies were eliminated from transmission and the connection was still established at the expense of lower throughput.

**Figure 4:**
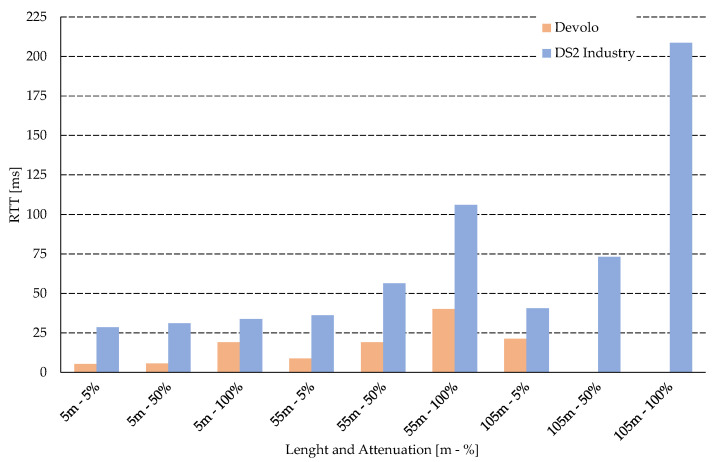
Round Trip Time (RTT).

Figure 4 shows the comparison of the RTT of the two modems. Devolo shows very low values, up to 50% of the noise are half the values for the distance of 5 and 55 m. The DS2 industrial modem is resistant to interference and shows high robustness as it was able to communicate in all defined scenarios, but shows significantly higher delay. This delay could issue in the case of real-time scenario, or using several’s streams with different priority.

The fault tolerance of measurement was verified with Iperf measurement. This measurement focused on fault tolerance caused by possible time variant conditions or different TCP windows size. The throughput with Iperf was measured for TCP layer with TCP window size 64 KB (the same as in RFC 6349). The results for the 24 h test are a median throughput of 94.05 Mbps with standard deviation of 1.73. This result shows stable conditions (the aim of laboratory measurement conditions with isolated power lines). Thanks to stable conditions, the system scalability is provided (repeatable environment for easy, time-efficient and low-cost evaluation of different BPL solutions and also results for comparison for the research community). Fault tolerance for RFC is given according to the phase of the RFC 6349 test. Before measurements, the MTU size and RTT were measured and according to the results the TCP windows size is computed. In this article, the fault tolerance of results was also verified via the field measurement.

## 7. Vision of The Proposed Integrated BPL Module

For the possibility of a greater use of broadband PLC technology in the environment of Smart Building and Smart Home, this article also presents a schematic design of a prototype of a broadband PLC modem integrated into an electrical outlet. The main advantage of this solution is a simple deployment in buildings by replacing actual electrical sockets. Current designs of PLC adapters used in home conditions are based on an adapter that plugs directly into an electrical outlet, which can be a problem, for example when controlling built-in appliances through a PLC adapter is needed. An example of a standard PLC adapter is presented in [47]. Another advantage is that, unlike PLC adapters, the integrated modem does not disturb the appearance of the socket and the possibilities of connecting devices.

The assumed transmission speed for reading the measured values with regard to the security of individual protocols should be at least 1 Mbps, for use within local networks with high-speed data transmission of at least 50 Mbps. For this reason, a Broadband PLC solution was considered for the integrated module instead of a Narrowband PLC, because the Narrowband PLC does not provide the necessary throughput.

**Figure 5:**
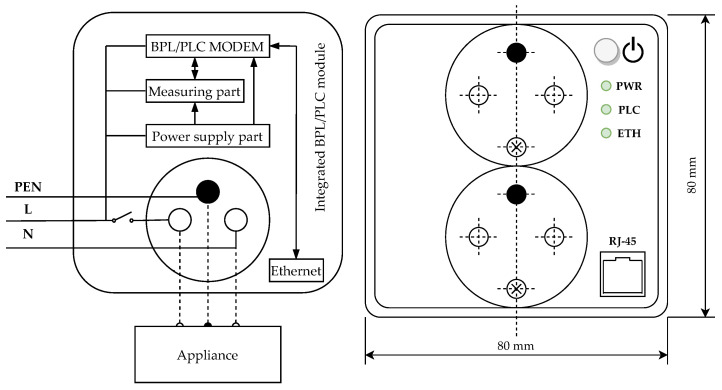
(**left**) Block diagram; and (**right**) Front panel of integrated BPL module.

Figure 5 (left) shows a block diagram of the whole module. The whole scheme consists of the following parts:

- The power supply part converts the AC (Alternating Current) mains voltage to the required lower DC (Direct Current) voltage and provides power to all parts of the integrated module.
- The measuring part is used to measure electrical parameters such as voltage and current and also allows switching an electrical outlet.
- The BPL/PLC modem allows Broadband communication via a power line with the end device (PC or other device).

Figure 5 (right) shows a front panel of the standard double electrical outlet with integrated BPL module. The front panel is equipped with a button for turning on or off the BPL modem, indicator diodes for monitoring the status of the modem, PLC and Ethernet communication lines. RJ-45 Ethernet connector is connected to the outside of the electrical outlet for connection of the end device.

**Figure 6:**
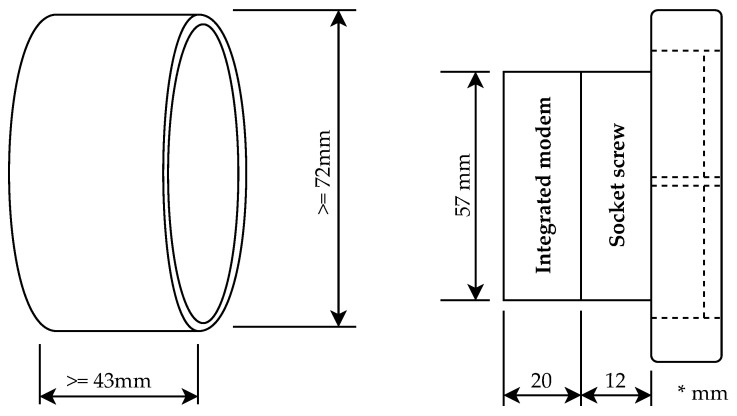
Wiring box and side view of integrated BPL module.

Figure 6 shows the minimum dimensions of the electrical wiring box for installing an integrated modem (left) and a side view of attaching the module to an electrical outlet socket screw (right). The prototype of the integrated modem was created for installation in circular electrical wiring boxes with a minimum depth of 43 mm and a diameter of at least 72 mm. The printed circuit board was designed with regard to the efficient use of space in the electrical wiring box. The total depth of the socket screw block with the installed integrated modem is 32 mm.

The authors of this article conducted market research and no company currently offers this type of device. During the analysis of patents, agreement was found with the patent in [48], which is, however, focused primarily on providing high speed data transmission through power line networks. The main difference of the device described above is its use, which is focused on power quality measurement of the electrical network, power consumption of the appliance, control, signalization and prediction of non-electrical failures in Smart Building applications. Design of the integrated PLC module described in this article was provided with a utility model protection.

## 8. Discussion

**Achievable throughput of BPL:** The throughput on the application layer is sufficient to fulfill the standard TR 61850–90–12 and to achieve throughput at the minimum of 2048 kbps. In the worst case scenario (noise level 11 dB), a disconnection could be seen, which will lead to a connection loss and the need to re-establish the connection and also re-establish the TLS connection. According to results in worst case noise scenario, the required availability of 99.9% cannot be fulfilled by the BPL technology. According to measurements, the throughput on the application layer of the G.hn standard is about 30 Mbps for a medium noise level. In the case of the worst case scenario, it is not possible to establish a connection. In the comparison of the G.hn design primary for in-home and industrial applications, the BPL modem is still able to establish a connection also in the worst case scenario with the throughput of only 3–4 Mbps.

**Possibilities for throughput measurement:** The TCP throughput methodology uses an algorithm that works with the so-called TCP window size, which it adapts through the whole test. In the case of RFC 6349, the size of the TCP window is determined when the test is initialized. This window determines the amount of data that can be transferred. Its size is therefore always related to the capacity of the network, which, as a rule, it must not exceed due to error-free and efficient delivery of messages. Furthermore, a receiver window is used, which must be optimized with respect to the performance of the receiver, in order to process the received data. Proper control of this window can therefore significantly affect the results of the measured communication. The TCP throughput methodology can be considered as the most accurate for measuring TCP/IP communication (but it is a non-standardized testing methodology, while RFC 6349 is the standard). The methodology is defined at a general level so that it is possible to generalize the measurement of data parameters and to extend the measurement from the physical layer of the network traffic. Thus, the technology (after slight modification) can be used to measure any transmission technology in general.

**Recommendation for achieve availability and security:** RFC 6349 represents TCP-based measurements which are necessary for TLS connections. The main advantage of the IETF method RFC 6349 is the fact that it uses the TCP protocol for the measurement itself, which is necessary for high availability and non-real-time communication. The disadvantage of using the TCP protocol is the need to set the transmit and receive windows and buffers correctly, to set the correct size of packets (frames) and that the actual data transmission can be affected by the need to retransmit erroneously transmitted packets (frames). The measured results may differ from the actual deployment with regard to the optimal settings and the software and performance of the end elements.

**Symmetrical and asymmetrical bandwidth allocation:** Dynamic bandwidth allocation (upload/download) can lead to a higher baud rate in asymmetrical use, i.e., a substantially higher baud rate in one of the directions, but it is not beneficial when used symmetrically.

**The limitations of our work:** For the measurements, these simplification were considered (potential future work):

- The noise generation only considered the frequency band of 1–50 MHz (G.hn considered the frequency band up to 100 MHz).
- The peak noise value cannot be set up.
- The methodology directly fit for PLC time variant condition is not provided.
- The topology with branches was not considered.
- The maximum length between modems for stable communication was not measured.

## 9. Conclusions

The best BPL solution for Smart Building applications must be identified and carefully selected. Not all BPL technologies are created equal and not all BPL technologies should be judged by the poor performance of some of them. The key parameters for evaluation of the communication performance of a Smart Building network are the data rate (throughput), robustness (availability and stability), noise immunity and communication distance without a repeater. The environment and set-up provided in this article enable the same tests to be repeated under the same conditions for all PLC technologies.

The article provides possible throughput of different BPL solutions for different conditions (noise and attenuation). For the G.hn standard, typically used in in-home applications, the throughput on the application layer was at least 70 Mbps for ideal noise scenario and at least 30 Mbps for medium worst noise scenario. The throughput for the worst case noise scenario was only 18 Mbps and only for a distance of 55 m. The results of laboratory measurements were confirmed via field measurements in a real power network. The throughput for IEEE 1901 standard was 20–22 Mbps. Currently, there are essentially two dominant standards, namely IEEE 1901 and ITU-T G.hn. Both standards offer speeds up to units of Gbps on the physical layer, and, according to measurements in this article, the throughput on application layer was only 104 Mbps).

For home use, HomePlug AV or AV2 PLC adapters are more widespread, but their throughput is limited. There are few vendors solutions for industry based on IEEE 1901, especially for medium voltage. The number of manufacturers offering G.hn or IEEE 1901 modems for industry is minimal, therefore this article also provides the vision and design of an integrated BPL model for industry.

The main contribution for research society is the repeatable topology considered for measurements, with which different standards or vendor solutions for BPL modems can be easily included for comparison.

The future work will be focused on extension of the topology (branches, power line length, noise and repeaters) while maintaining repeatability. In addition, research of methods for throughput testing focusing directly on PLC will be done.

## Figures and Tables

**Table 1:** Summary of related works.

No.	Authors	Year	Method Used	Layer	Standard	Throughput
[18]	G. López et al.	2019	theoretical	PHY max	HP AV	200 Mbps
[19]	Hashiesh, Fahd	2009	measurement	PHY	Corinex	12–26 Mbps
[20]	Horvat, Balkić, Zadar	2012	measurement	Transport	HP AV	30–35 Mbps
[21]	Castor, Natale, Silva, Segatto	2014	measurement	PHY	undef	50–120 Mbps
[22]	Tomimura, Neto	2008	measurement	Transport	HP AV	5.8–21 Mbps
[23]	Cui, Lio, Cao, Xu	2018	simulation	PHY	undef	up to 1 Mbps
[24]	Sangsuwan et al.	2014	measurement	Transport	HP GP	0.88–3.3 Mbps
[25]	Lee et al.	2003	measurement	Application	HP AV	1.6–5.3 Mbps
[26]	T. Matsuo, S. Maekawa	2005	measurement	PHY	CE Marking	45–100 Mbps
[27]	Schwager et al.	2005	simulation	PHY	undef	190.6 Mbps
[28]	Piñero et al.	2014	simulation	Transport	HP AV	10–75 Mbps
[29]	Nico Weling, Neda Nazari	2011	measurement	PHY	HP AV	11 Mbps
[30]	S. Sasikumar, S. Narayanan	2015	simulation	PHY	undef	180 Mbps
[31]	Moises V. Ribeiro et al.	2015	measurement	Application	undef	1.23–5.15 Mbps
[32]	Anton. G. Merkulov et al.	2019	measurement	Transport	HP AV	about 24 Mbps
[33]	Fujdiak et al.	2018	measurement	PHY	HP AV2	800–900 Mbps
[34]	Mizutani et al.	2011	measurement	PHY	HD-PLC	over 63 Mbps
[35]	Orgon et al.	2019	measurement	Transport	HP AV2	622–766 Mbps
[36]	Osman, Nisar, Altrad	2014	measurement	Transport	HP AV	95.14 Mbps
[37]	Arab, Karimi, Safavi	2016	simulation	Application	HP AV	23 Mbps
[38]	Hallak, Berners and Mengi	2020	measurement	Transport	ITU-T G.hn	95.5 Mbps
[39]	IEEE standard	2019	theoretical	PHY	IEEE	>100 Mbps
[40]	IEEE standard	2018	measurement	Transport	EV PLC	48 Mbps

**Table 2:** Basic description and parameters of power line.

Operating Voltage	0.4 kV
Type of power line	underground power line
Material	AYKY
Conductor cross-section	3 × 185 + 95
Length according to GIS	104.76 m
Year of construction	1992

**Table 3:** Achieved results of RFC 6349 methodology.

Direction	TCP Window	Ideal L4 (Mbps)	Actual L4 (Mbps)	TCP Efficiency (%)	Buffer Delay (%)	Minimum RTT (ms)
L → R	37.9 KB (2 conn. @ 18.4 KB)	93.9	20.9	100	379.53	3.235
R → L	37.9 KB (2 conn. @ 18.4 KB)	93.9	22.2	100	350.99	3.235

**Table 4:** Tested broadband PLC adapters.

**Manufacturer:**	Cisco	Zyxel	Devolo	DS2 Industry	RAKwireless
**Model:**	PLE500	PLA5206	Magic 2	-	LX200V30
**Chipset**	QCA7450	BCM60333	88LX5152	DSS95X	AR7420
**Standard:**	HP AV2	HP AV2	G.hn	DS2	HP AV
**Bandwidth [MHz]:**	2–68	2–86	2–100	2–34	2–68
**PHY speed [Mbps]:**	600	1000	2400	200	500
**ETH Interface [Mbps]:**	1000	1000	1000	100	100
**Security:**	128b AES	128b AES	128b AES	256b AES	186b AES

**Table 5:** Promax PROPOWER–1 specifications.

Promax PROPOWER–1	
**Frequency range:**	1–50 MHz
**Output power:**	20 dBm (−47 dBm/Hz) ± 2 dBm
**Frequency response:**	± 1.5 dB
**Variable attenuator:**	0 to 10 db
**Output impedance:**	50 Ω

## Data Availability

Author do not will to publish data online.
